# Rare Encounter of Renal Hydatid Cyst: A Case Report

**DOI:** 10.31729/jnma.6994

**Published:** 2021-07-31

**Authors:** Prabina Basnet, Sudeep Chapagain, Rasik Neupane, Abishkar Thapa

**Affiliations:** 1 School of Medicine, Patan Academy of Health Sciences, Lagankhel, Lalitpur, Nepal; 2 Kathmandu Hospital, Tripureshwor, Kathmandu, Nepal; 3 Sukraraj Tropical and Infectious Disease Hospital, Teku, Kathmandu, Nepal

**Keywords:** *Echinococcus granulosus*, *hydatid cyst*, *laparoscopic*

## Abstract

Cystic Echinococcosis or Hydatid disease is caused by the infection with the larval stage of long tapeworm, Echinococcus granulosus. This condition often remains asymptomatic for years before the cyst grows large enough to cause symptoms in affected organs. The most common organs involved are liver and lungs although the heart, brain, bone, central nervous system, and kidney may also be involved. This case is about a young woman who presented with left flank pain and urinary tract infection who was later diagnosed as having left renal hydatid cyst. The cyst was approximately 7.8*6.6*8cm with internal multiple septations at the lower pole cortex of the left kidney. Laparoscopic pericystectomy was performed and with no postoperative complications, she was discharged on albendazole and other supportive medication. With timely management using combination therapy, this condition is curable and the patient can live a healthy life with normal kidney function.

## INTRODUCTION

Echinococcosis/Hydatidosis is a zoonotic parasitic disease caused by larval stages of tapeworm, mainly Echinococcus granulosus. The larval stage develops in intermediate hosts including man and adult stage is found in carnivores. Clinical disease results mainly from pressure effects caused by the enlarging cyst several years after initial infection.^1^

CT scan is superior to ultrasonography with higher diagnostic accuracy for diagnosis. Serology and other tests are considered complementary.^2^ First line treatment is surgical removal. Albendazole (10-15mg/kg/day orally) is prescribed before and after surgery. We report a case of 24 years female, presented with abdominal pain and diagnosis of left renal hydatid cyst.

## CASE REPORT

24-year-old female from Simikot, Humla presented to Patan hospital with left flank pain and feverish feeling for 5 days. Vitals were stable and the only notable finding was tenderness over the left hypochondrium on deep palpation. Urinalysis revealed pus cells with plenty of RBCs without growth on culture. Biochemistry and eosinophil count was normal with slightly increased total count. No hydatiduria was noted in urinalysis.

**Figure 1:**
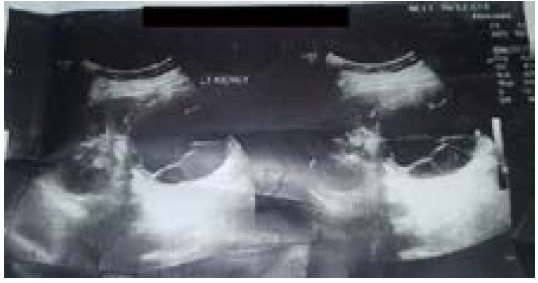
Ultrasound image showing left renal hydatid cyst with internal septations.

Ultrasonography revealed cystic lesion measuring 7.1cm x 7.9cm in lower pole cortex of left kidney with internal septations suggestive of hydatid cyst (Gharbi Type III) (Figure 1).

**Figure 2:**
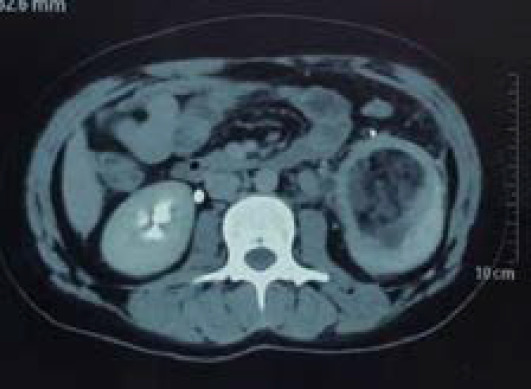
CT image showing cystic lesion in left kidney with internal septations.

Contrast enhanced computed tomography scan further supported the findings of USG. CT scan revealed cystic lesion measuring 7.8 x 6.6 x 8.0cm at mid and lower pole cortex of left kidney with notable internal multiple septations. (Figure 2). The postoperative period was uneventful. She was discharged on anthelmintic (albendazole) and other medications with regular follow up.

Management of the UTI was done with follow up for the cyst. DTPA scan was carried out in the next follow which showed a normal sized left kidney with impaired function and delayed non obstructive clearance evidenced by differential function of 36 and GFR 28.8ml/min/1.73sqm.

Laparoscopic pericystectomy for the left hydatid cyst was performed. With intraoperative findings of a big hydatid cyst in the inferior pole of the left kidney measuring approximately 8.0 x 7.0 x 8.0cm containing multiple daughter cyst and pus.

## DISCUSSION

Hydatid cyst, the larval stage of Echinococcus, is a bladder-like cyst formed in various organs and tissues following the growth of the oncospheres of an Echninoccus tapeworm in that specific organ or tissue. Oncospores developed from eggs later develop to hydatid cyst (infective stage) in intermediate host including man. Infection is through swallowing eggs from contaminated vegetables or uncovered foods or inhalation of dust with eggs. After ingestion by the intermediate host, the oncosphere penetrates the gut wall and circulates in the blood (portal circulation) to the liver or in the lymph to the lungs (commonest sites) but sometimes escape into the general systemic circulation and develop in other organs and tissues like kidney.^1^

A World Health Organization (WHO) study in 2010 estimated the incidence of cystic echinococcosis per 100,000 population in Southeast Asia to be 0.8.^3^ Renal echinococcosis usually presents between the third and fifth decade of life and consists of 2-3% of all cases.^4^ It usually remains asymptomatic for many years.^5^ Symptoms and signs of hydatid cyst disease depend on the involved organ, the site, and the secondary spread. A palpable mass is the most common clinical finding. It may present with lower back pain, hematuria, or an abdominal lump.^6,7^ In up to 18 to 20% of cases, the rupture of the cyst into the urinary collecting system causes hydatid urea which is a pathognomonic sign of renal hydatidosis.^8,9^

Combination of diagnostic methods is necessary for diagnosis of hydatid cyst in some organs with no symptoms (e.g. kidney) with clinical history, serological analysis, urine analysis and medical imaging. Specific serological tests could be done using immunoglobulin G enzyme-linked immunosorbent assay and immunoelectrophoresis.^10^ Radiologic imaging through ultrasonography (US), computed tomography (CT), and magnetic resonance imaging (MRI) proves the diagnosis.^9^

For treatment of these cases both pharmacotherapy and surgery are needed. Surgery is via open and retroperitoneal approach.^11,12^ When possible, kidney sparing cyst removal is performed through cystectomy and pericystectomy; however, nephrectomy is needed when the hydatid cyst invades a major renal part or in cases of hydatiduria.^9^ Albendazole is the antiparasitic agent of choice whether prescribed alone or as adjuvant therapy in two daily doses adding up to 10-15mg/kg/day. Albendazole in cycles of three to six months to treat hydatid disease with liver involvement induces cyst shrinkage and inactivation according to WHO-Informal Working Groups on Echinococcosis (WHO-IWGE) standards based on ultrasound characteristics, although associated with frequent recurrence. Drug therapy for renal hydatid disease is safe but ineffective. Therefore, surgery has been considered the treatment of choice. Albendazole is prescribed as a prophylactic drug at a dosage of 10-15mg/kg/day (maximum of 400 mg) orally before and after surgery.^6^

In our case, laparoscopic pericystectomy was performed for the left renal hydatid cyst. The kidney was preserved as the major renal parts were not involved. The postoperative period was uneventful. The patient completed five cycles of albendazole therapy and now she is asymptomatic and living a healthy life.

Although a rare entity, renal hydatid cyst can be encountered in our setting. It can be found incidentally while evaluating for other common urological conditions like urinary tract infection (UTI). Following diagnosis, management using a combination of surgical and medical treatment can help patient with this condition to continue a disease free and healthy life.
